# Editorial: IBD Management—Novel Targets and Therapeutic Perspectives

**DOI:** 10.3389/fphar.2020.00448

**Published:** 2020-04-21

**Authors:** Luca Antonioli, Matteo Fornai, Barbara Romano, Carolina Pellegrini, Corrado Blandizzi

**Affiliations:** ^1^ Department of Clinical and Experimental Medicine, University of Pisa, Pisa, Italy; ^2^ Department of Pharmacy, School of Medicine and Surgery, Univeristy of Naples Federico II, Naples, Italy; ^3^ Department of Pharmacy, University of Pisa, Pisa, Italy

**Keywords:** IBD, immune cells, novel therapeutic agents, inflammatory resolution, cytokines

Inflammatory bowel diseases (IBDs) are a group of idiopathic, chronic, relapsing, inflammatory conditions, which include ulcerative colitis and Crohn’s disease (Jairath and Feagan, 2020). Collectively, these disorders are characterized by debilitating physical and psychosocial symptoms, such as fever, weakness, and weight loss and are accompanied by intestinal symptoms associated with chronic inflammation of the intestinal mucosa, such as abdominal pain and chronic diarrhea (Jairath and Feagan, 2020). Although the etiology of IBD remains unknown, it is considered to result from an abnormal immune response against environmental factors, including luminal and microbial antigens, in genetically susceptible hosts (Jairath and Feagan, 2020).

For several decades, medical treatments of IBDs were limited to nonbiological therapies (*i.e.*, aminosalicylates, thiopurines, and steroids), which, despite providing symptomatic relief, affected scarcely the disease course (Kim and Cheon, 2017). The increasing advances in understanding the molecular mechanisms underlying the pathogenic mechanisms involved in IBD then paved the way toward novel therapies aimed at curbing the activity of proinflammatory cytokines pivotally involved in the onset and progression of IBDs (Pagnini et al.).

An interesting Review by Pagnini et al. included in our Research Topic provides an exhaustive overview of the therapeutic armamentarium currently available for the management of patients with IBDs. At present, beyond the introduction of antitumor necrosis factor (TNF) biodrugs (infliximab, adalimumab, and certolizumab), which reduced the need for surgery and hospitalization and improved significantly the quality of life of patients, novel biological therapies aimed also at counteracting other proinflammatory cytokines (*i.e.* IL-12/IL-23, IL-6, IL-13, and IL-17) are currently available or under active evaluation in advanced clinical trials (Pagnini et al.). The introduction into the clinical practice of Janus kinase inhibitors (JAK inhibitor; tofacifinib), represents undoubtedly an intriguing pharmacological strategy designed to ablate simultaneously the downstream signaling of several cytokine receptors (Pérez-Jeldres et al.).

In parallel, with the above anticytokine pharmacological approaches, another strategy has been directed towards the development of selective lymphocyte trafficking inhibitors (*i.e.*, biological drugs vedolizumab and etrolizumab), and the antimucosal vascular addressin cell adhesion molecule-1 (anti-MAdCAM-1; the small molecule PF-00547659) agent. In particular, Lichnog et al. reported that treatment with etrolizumab induced the internalization of *α*4*β*7 integrin, and that this event functionally impaired *β*7-dependent cellular adhesion to MAdCAM-1. Of note, the authors demonstrated that the internalization of *α*4*β*7 integrin was higher with etrolizumab as compared with vedolizumab (Lichnog et al.). Likewise, the trafficking of lymphocytes represents another interesting progress that can now be targeted by a small molecule (Pérez-Jeldres et al.). Sphingosine-1-phosphate receptor agonism, operated *via* fingolimod, KRP-203, ozanimod, etrasimod or amiselimod is another novel strategy that acts, in part, by interfering with lymphocyte recirculation, through the blockade of lymphocyte egress from lymph nodes (Pérez-Jeldres et al.).

In recent years, the perspective for innovative IBD therapies is changing. Indeed, it is emerging that novel pharmacological approaches to IBD management are refocusing their attention toward the modulation of the interplay between the innate and the adaptive components of the immune system (Vadstrup and Bendtsen, 2017; Bassoy et al., 2018; Stojanovic et al., 2018). In this context, the natural killer group 2, member D (NKG2D) receptor is emerging as an attractive target in IBDs. The NKG2D receptor is a type II transmembrane protein expressed by both innate and adaptive immune cells, including natural killer (NK) cells, CD8^+^ T cells, invariant NKT cells, *γδ* T cells, and some CD4^+^ T cells under certain pathological conditions (Stojanovic et al., 2018). In particular, when activated, both macrophages and DCs upregulate NKG2D, thereby enabling them with additional mechanisms for regulating lymphocyte responses (Mao and Rieder, 2019). On this basis, blocking NKG2D would be another new mechanism of action for moderate to severe CD patients, as highlighted by the evidence about a significant increase in clinical remission in CD patients treated with an anti-NKG2D antibody (Vadstrup and Bendtsen, 2017).

The IL-36 cytokine family, produced predominantly by epithelial cells, acts on several cells including the immune cells, epithelial cells, and fibroblasts and is increased in IBD patients, thus representing another interesting target to manage bowel inflammation (Bassoy et al., 2018). In this regard, anti-IL36R antibodies are entering phase II trials in patients with moderate to severe ulcerative colitis (UC) (Mao and Rieder, 2019).

The termination of inflammation is governed by endogenous molecular factors collectively referred to as ‘mediators of resolution’ of inflammation. There is now strong evidence to suggest that failed resolution may underpin autoimmune and inflammatory diseases, including IBDs, and could thus be targeted to curb inflammation (Sugimoto et al., 2019). The field of resolution pharmacology represents an intriguing way worthy of being pursued for the management of inflammatory disorders, changing the paradigm of ‘fighting inflammation’ to ‘targeting and advancing inflammation resolution’ (Sugimoto et al., 2019). Over the last few years, increasing efforts have been addressed toward the characterization of proresolving mediators, allowing to identify novel molecular targets useful to design resolution-based therapies for IBDs (Sugimoto et al., 2019).

The ways forward for the resolution of inflammation can be different. Several authors have identified the antisense oligonucleotide technology as a specific, rapid, and potentially high-throughput approach (Di Fusco et al.; Scarozza et al.).

It is also emerging that the hallmarks of mitochondrial dysfunction, including oxidative stress and altered ATP production, are evident in the intestines of patients with IBD (Novak and Mollen, 2015). In this regard, it is widely acknowledged that the mitochondria are capable of regulating the proinflammatory responses of cells through the activation of a molecular complex known as the NLRP3 inflammasome (Novak and Mollen, 2015). Recently, Pellegrini et al. showed that direct NLRP3 inhibition can be a suitable strategy for the treatment of bowel inflammation. Indeed, INF39, a novel NLRP3 inhibitor, was found to be more effective than caspase-1 inhibition or IL-1*β* receptor blockade in reducing systemic and bowel inflammatory alterations in DNBS-colitis (Pellegrini et al.).

Overall, this Research Topic is providing new insights into novel pharmacological entities that are already present or are facing the therapeutic landscape for the management of IBDs. These range from innovative antibodies or small molecules aimed at stemming inflammatory cytokines pivotally involved in the IBD pathophysiology to strategies aimed at disrupting the vicious circle that occurs among cells of the innate and acquired immunity, as well as to intriguing approaches aimed at correcting defective function of proresolution mechanisms to rectify chronic inflammatory conditions. If successful, the impact of all these approaches will improve significantly not only the management of IBDs but also the quality of life of individuals suffering from these disorders.

## Author Contributions

AL, FM, BR, CP and CB participated to write the Editorial.

## Conflict of Interest

The authors declare that the research was conducted in the absence of any commercial or financial relationships that could be construed as a potential conflict of interest.

After manuscript acceptance, the author list of this article was amended to include an additional author. The Handling Editor AAI acknowledges a shared affiliation and past co-publications with this added author BR. This was declared to the Editorial Office during article production. The review process met the standards of a fair and objective review.
